# Correction: The impact of digital economy on the upgrading of manufacturing structure

**DOI:** 10.1371/journal.pone.0320514

**Published:** 2025-03-17

**Authors:** Ting Chen, Songlan Zhou

There is an error in affiliation 1 for author Ting Chen. The correct affiliation 1 is: College of Digital economy and Trade, Guangzhou Huashang College, Zengcheng District, Guangzhou City, Guangdong Province, China.
