# Supplementary material for: The impact of digital economy on the upgrading of manufacturing structure
Source: PLoS One. 2024 Jul 25;19(7):e0307184. doi: 10.1371/journal.pone.0307184 (PMC11271849; doi:10.1371/journal.pone.0307184)
Supplement: S1 Data — (PDF) [file pone.0307184.s001.pdf]

prov	year	manuh	inv	open	gov	pgdp	urban	mark	intt	digital	hum	manuhigh
东 莞	2012	0.58708	12.7839	1.8103	0.0174605	5.7632721	0.8867	0.9216	0.7137229	-0.492988	393	0.50957
东 莞	2013	0.61428	7.86352	1.7183	0.0190862	6.0842927	0.8875	0.9332	0.6833605	-0.607305	346	0.53833
东 莞	2014	0.63586	17.5045	1.6972	0.0212285	6.3064767	0.8881	0.9387	0.6365563	-0.795183	607	0.5609
东 莞	2015	0.63754	36.1617	1.5604	0.0872034	6.6810373	0.8914	0.9434	0.5579854	-0.80414	550	0.56976
东 莞	2016	0.67904	9.60987	1.5715	0.0825364	7.2027529	0.8934	0.9494	0.4860377	-0.550733	339	0.61633
东 莞	2017	0.67948	8.616	1.5174	0.0826381	7.863736	0.8944	0.9571	0.4307648	-0.396545	343	0.61995
东 莞	2018	0.68993	9.83014	1.5219	0.0867998	8.470843	0.8993	0.9586	0.3840019	0.0324002	378	0.63399
东 莞	2019	0.67862	39.939	1.4602	0.0910884	9.069606	0.9031	0.9547	0.3549086	0.3038027	598	0.62467
东 莞	2020	0.68097	81.0657	1.3635	0.0861274	9.3194102	0.9215	0.9543	0.3411926	0.4965974	788	0.62062
东 莞	2021	0.65058	126.921	1.4045	0.0812993	10.328391	0.9224	0.9473	0.3529772	-0.115194	1842	0.58098
中 山	2012	0.55312	3.59482	0.865	0.1576194	6.6859655	0.8792	0.9313	0.5635075	-0.552511	72	0.47296
中 山	2013	0.56209	3.14661	0.832	0.1676735	6.7972599	0.88	0.9211	0.5857731	-0.537472	77	0.47327
中 山	2014	0.5896	3.18545	0.8042	0.1621256	6.7954916	0.8807	0.9133	0.5727451	-0.515078	77	0.48124
中 山	2015	0.5989	4.57225	0.8152	0.1310669	6.9128615	0.8741	0.9168	0.5389676	-0.480258	76	0.49195
中 山	2016	0.59825	5.35505	0.7902	0.1298637	7.0351834	0.8732	0.914	0.5223544	0.047437	80	0.48826
中 山	2017	0.6315	5.34542	0.8784	0.1548619	7.1198158	0.8724	0.9047	0.5118042	0.0765985	81	0.5162
中 山	2018	0.63237	4.476	0.7668	0.1434049	7.2118893	0.8715	0.9169	0.4888648	0.3642646	58	0.52008
中 山	2019	0.62898	6.29772	0.7642	0.131808	7.2013999	0.8706	0.9158	0.4322809	0.7530869	66	0.50484
中 山	2020	0.64918	7.01632	0.6926	0.1177854	7.2329359	0.8696	0.9304	0.3975453	0.6412941	70	0.5429
中 山	2021	0.63315	9.25474	0.7557	0.1324893	8.0156598	0.87	0.9308	0.4278739	0.3453271	111	0.53159
佛 山	2012	0.45231	5.35891	0.5858	0.0659588	8.5288993	0.9487	0.9579	0.3365739	-0.49773	160	0.37018
佛 山	2013	0.44271	5.93348	0.5649	0.0696645	8.7193626	0.9488	0.953	0.3237145	-0.528015	137	0.3642
佛 山	2014	0.44917	7.04308	0.568	0.070551	8.9742466	0.9489	0.955	0.3139559	-0.529758	122	0.3741
佛 山	2015	0.45589	2.11178	0.5041	0.0986642	9.4607721	0.9428	0.9553	0.3143492	-0.601765	145	0.38075
佛 山	2016	0.47044	6.11955	0.4689	0.0794684	10.069874	0.9433	0.9568	0.3137589	-0.671941	104	0.39142
佛 山	2017	0.45222	6.19685	0.4645	0.0825993	10.572875	0.9436	0.9515	0.305485	-0.63829	109	0.36947
佛 山	2018	0.46706	5.91616	0.461	0.0808422	10.927221	0.945	0.9552	0.2911657	-0.225825	92	0.38106
佛 山	2019	0.47848	55.1746	0.4496	0.0876481	11.491413	0.9467	0.9554	0.2837665	-0.017273	249	0.38561
佛 山	2020	0.49455	136.777	0.4704	0.0932325	11.354498	0.952	0.9532	0.2848163	0.0247592	719	0.40759
佛 山	2021	0.49372	147.281	0.5068	0.0881839	12.708467	0.9521	0.9483	0.259464	0.6214656	899	0.40133
广 州	2012	0.61659	429.545	0.5458	0.0991532	9.5549628	0.8502	0.7269	0.6223971	0.5316938	12603	0.46159
广 州	2013	0.61755	427.274	0.4752	0.089444	10.423547	0.8527	0.7255	0.6165366	1.266237	13425	0.46199
广 州	2014	0.64024	452.024	0.4801	0.085966	10.752786	0.8543	0.7219	0.612333	1.157286	13407	0.48399
广 州	2015	0.66748	524.946	0.4788	0.0995955	11.105979	0.8422	0.7544	0.6050119	0.5220057	13260	0.50638
广 州	2016	0.67476	544.472	0.4602	0.1047294	11.33997	0.8435	0.7789	0.5810272	0.4632661	13801	0.52732
广 州	2017	0.69652	586.647	0.4889	0.1100064	11.60508	0.8441	0.6486	0.5461944	0.4643704	15148	0.57116
广 州	2018	0.69683	532.561	0.4672	0.119328	11.851057	0.8475	0.6437	0.5259055	1.227444	15021	0.57919
广 州	2019	0.67419	605.141	0.4195	0.1201664	13.139962	0.8513	0.6357	0.545157	1.406325	16221	0.56508
广 州	2020	0.69357	670.859	0.3802	0.117782	13.531512	0.8619	0.6213	0.5249225	1.613681	18048	0.58825
广 州	2021	0.68379	769.221	0.3834	0.1070129	15.03664	0.8646	0.5559	0.5049946	2.211097	19194	0.57901
惠 州	2012	0.63759	9.88936	1.313	0.0737658	4.7734131	0.639	0.7057	0.6305648	-1.416965	376	0.54299
惠 州	2013	0.65362	9.82017	1.314	0.0762618	5.2210905	0.66	0.7494	0.6372695	-1.26003	371	0.56929
惠 州	2014	0.67039	10.1518	1.2164	0.0899954	5.5899771	0.67	0.7921	0.6356322	-1.224123	363	0.57154
惠 州	2015	0.67728	10.3788	1.0925	0.1572929	5.6801893	0.6441	0.8411	0.614245	-1.175454	346	0.59001
惠 州	2016	0.66897	14.0074	0.9064	0.1515335	6.0361146	0.6508	0.8525	0.5820195	-1.011261	296	0.57424
惠 州	2017	0.69179	18.3613	0.9119	0.1479036	6.6007332	0.6567	0.8252	0.5788295	-0.837914	305	0.57858
惠 州	2018	0.70323	13.0122	0.833	0.1359418	6.9205511	0.6761	0.7321	0.5324315	-0.383273	303	0.58524
惠 州	2019	0.68176	14.3235	0.6462	0.1466421	7.0949354	0.6961	0.7365	0.468829	0.0218975	337	0.55688
惠 州	2020	0.70301	31.3643	0.5809	0.1487898	7.1220332	0.728	0.8168	0.4692647	0.1851471	668	0.58695
惠 州	2021	0.68931	51.7076	0.6138	0.1332655	8.2112974	0.729	0.8186	0.4402057	0.1357557	666	0.56626
江 门	2012	0.43119	6.35514	0.6302	0.1145055	4.214778	0.632	0.8924	0.5197522	-1.582496	146	0.30647
江 门	2013	0.39537	5.61129	0.611	0.1186097	4.4717719	0.641	0.897	0.5165805	-1.442196	128	0.27645
江 门	2014	0.44151	5.2009	0.6009	0.1255338	4.6176854	0.642	0.9119	0.5060613	-1.126033	121	0.27886
江 门	2015	0.44861	7.27545	0.5413	0.1287894	4.9767075	0.6353	0.9081	0.504964	-1.085148	127	0.28233
江 门	2016	0.44043	6.81087	0.5086	0.1181852	5.3941017	0.6385	0.9216	0.4899284	-0.80155	135	0.29419
江 门	2017	0.42026	7.22179	0.5044	0.1213669	5.9244416	0.6413	0.9129	0.4796004	-0.650596	135	0.3208
江 门	2018	0.47668	10.4511	0.4905	0.1259078	6.4163406	0.6512	0.9075	0.4780271	-0.199151	135	0.29231

江门	2019	0.41797	12.621	0.4525	0.1337177	6.6621948	0.6604	0.8951	0.4443772	-0.14876	140	0.31108
江门	2020	0.43962	7.79334	0.4461	0.1381141	6.7026729	0.6763	0.8937	0.410879	-0.163205	170	0.33754
江门	2021	0.45513	17.6513	0.4969	0.1278005	7.4721563	0.6784	0.8866	0.3980782	-0.034236	172	0.36452
深圳	2012	0.78532	29.2614	2.2717	0.1209583	11.640734	1	0.8565	0.5540244	-0.027783	970	0.74808
深圳	2013	0.78221	25.0412	2.2843	0.1160273	12.420807	1	0.8487	0.5206158	0.6146891	1035	0.74348
深圳	2014	0.78654	31.0108	1.8723	0.1353711	13.044781	1	0.8529	0.4872625	0.6931723	1746	0.74969
深圳	2015	0.81585	33.0546	1.4925	0.1910127	13.527105	0.9981	0.8594	0.4470356	0.5138074	1915	0.78183
深圳	2016	0.83546	37.6215	1.2717	0.2035722	14.249368	0.9985	0.8651	0.4120574	0.648533	1435	0.80027
深圳	2017	0.8382	44.8078	1.2038	0.1973259	15.073894	0.998	0.8843	0.3694874	0.4608994	1206	0.80421
深圳	2018	0.84427	88.6774	1.1873	0.1694984	15.531963	0.9982	0.9068	0.3464599	1.125077	1955	0.81405
深圳	2019	0.83412	102.812	1.1033	0.1686676	15.988252	0.9952	0.9078	0.3028088	1.529448	2756	0.80761
深圳	2020	0.8479	300.771	1.0991	0.1505249	15.982023	0.9954	0.9105	0.2869086	1.797853	3827	0.82032
深圳	2021	0.83585	283.199	1.1556	0.1490378	17.366279	0.9981	0.8858	0.3125746	2.544075	5576	0.80224
珠海	2012	0.75635	6.59036	1.9106	0.1406017	9.358882	0.8782	0.7262	0.6171336	0.1221854	161	0.60894
珠海	2013	0.75952	10.0791	2.0026	0.1501073	10.093896	0.8785	0.725	0.5909225	0.508948	162	0.6367
珠海	2014	0.7747	9.93559	1.8081	0.1477578	10.98456	0.8787	0.7051	0.5640177	0.7123678	106	0.6591
珠海	2015	0.79924	10.936	1.3364	0.1753953	11.787894	0.8813	0.6949	0.4908473	1.029109	100	0.69761
珠海	2016	0.79232	30.6644	1.1229	0.1700882	12.716718	0.8825	0.6869	0.4554875	1.138895	220	0.69743
珠海	2017	0.75262	42.179	1.0165	0.1677712	14.609583	0.8834	0.7646	0.4886549	1.015549	270	0.62647
珠海	2018	0.75286	32.7211	1.0096	0.1779792	15.034491	0.8879	0.7385	0.4711247	1.254815	303	0.63065
珠海	2019	0.73311	148.729	0.8445	0.1787741	15.170165	0.8921	0.8652	0.4488239	1.69559	211	0.59858
珠海	2020	0.73287	249.726	0.7768	0.1925996	14.71645	0.9047	0.8784	0.4215253	1.509301	222	0.61028
珠海	2021	0.73324	286.57	0.8552	0.2026554	15.791352	0.9075	0.8647	0.4276635	2.051262	227	0.60237
肇庆	2012	0.28835	4.88468	0.2732	0.1310015	3.240304	0.4262	0.9126	0.3822769	-1.825126	156	0.18225
肇庆	2013	0.27657	5.35739	0.2597	0.13063	3.6170719	0.4382	0.9183	0.3750444	-1.662489	158	0.17505
肇庆	2014	0.27469	4.19037	0.2607	0.1426714	3.9624911	0.4401	0.9259	0.3642759	-1.553979	153	0.1761
肇庆	2015	0.27859	4.59555	0.3023	0.1582347	4.233287	0.4433	0.933	0.3593547	-1.533775	148	0.17005
肇庆	2016	0.28729	5.70429	0.2529	0.1370544	4.5151003	0.4483	0.9341	0.3738998	-1.282281	146	0.17285
肇庆	2017	0.27967	5.38353	0.1821	0.1379922	4.8780864	0.453	0.9101	0.3195056	-1.141248	137	0.16946
肇庆	2018	0.2608	8.21413	0.1854	0.1501788	5.1879034	0.4699	0.9046	0.3030533	-0.877333	139	0.16718
肇庆	2019	0.26227	10.9386	0.1797	0.1562421	5.5175741	0.4852	0.9099	0.2606651	-0.646591	144	0.17068
肇庆	2020	0.2729	11.4309	0.1786	0.186139	5.6356581	0.5102	0.8954	0.2389424	-0.617366	144	0.18958
肇庆	2021	0.29433	13.5463	0.153	0.1497363	6.4268581	0.5191	0.8962	0.2160119	-0.610449	175	0.2171
