# Supplementary material for: The impact of digital economy on the upgrading of manufacturing structure
Source: PLoS One. 2024 Jul 25;19(7):e0307184. doi: 10.1371/journal.pone.0307184 (PMC11271849; doi:10.1371/journal.pone.0307184)
Supplement: Attachment — Submitted filename: PONE-D-24-01707_reviewer.pdf [file pone.0307184.s002.pdf]

PLOS ONE

The impact of digital economy on the upgrading of manufacturing structure

--Manuscript Draft--

**Manuscript Number:**	PONE-D-24-01707
**Article Type:**	Research Article
**Full Title:**	The impact of digital economy on the upgrading of manufacturing structure
**Short Title:**	Empirical evidence from the Pearl River Delta, China
**Corresponding Author:**	Ting Chen<br>guangzhou huashang xueyuan: Guangzhou Huashang College<br>Guangzhou, CHINA
**Keywords:**	Digital economy, The upgrading of manufacturing structure, Endogenous economic growth model, Mediating effect model
**Abstract:**	The global economic situation is in a downturn, and the upgrading of manufacturing structure is a necessary transformation path for the manufacturing industry to achieve high-speed and stable development. The article analyzes the theoretical mechanism of the digital economy affecting the upgrading of manufacturing structure through the endogenous economic growth model, constructs a three-stage mediation effect model, and empirically researches the path of the digital economy affecting the upgrading of manufacturing structure in the Pearl River Delta. The study finds that the digital economy has a significant positive promoting effect on the upgrading of manufacturing structure. In terms of the influencing mechanism, the enhancement of the level of independent innovation and the advancement of the human capital structure are the important paths of the digital economy in promoting the upgrading of manufacturing structure. in addition, the digital economy is negatively correlated with the import of technology, and the import of technology is not an important path for the digital economy to promote the upgrading of manufacturing structure. The research results also find that government support is indispensable to the upgrading of manufacturing structure, and government deployment and control are conducive to accelerating the process of the upgrading of manufacturing structure.
**Order of Authors:**	Ting Chen
	Songlan Zhou
**Additional Information:**	
**Question**	**Response**
**Financial Disclosure**<br><br>Enter a financial disclosure statement that describes the sources of funding for the work included in this submission. Review the submission guidelines for detailed requirements. View published research articles from *PLOS ONE* for specific examples.<br><br>This statement is required for submission and **will appear in the published article** if the submission is accepted. Please make sure it is accurate.	Yes

Powered by Editorial Manager® and ProduXion Manager® from Aries Systems Corporation

**Funded studies**<br>Enter a statement with the following details:<br>• Initials of the authors who received each award<br>• Grant numbers awarded to each author<br>• The full name of each funder<br>• URL of each funder website<br>• Did the sponsors or funders play any role in the study design, data collection and analysis, decision to publish, or preparation of the manuscript?<br><br>Did you receive funding for this work?	
Please add funding details.<br>as follow-up to "**Financial Disclosure**<br><br>Enter a financial disclosure statement that describes the sources of funding for the work included in this submission. Review the submission guidelines for detailed requirements. View published research articles from *PLOS ONE* for specific examples.<br><br>This statement is required for submission and **will appear in the published article** if the submission is accepted. Please make sure it is accurate.<br><br>**Funded studies**<br>Enter a statement with the following details:<br>• Initials of the authors who received each award<br>• Grant numbers awarded to each author<br>• The full name of each funder<br>• URL of each funder website<br>• Did the sponsors or funders play any role in the study design, data collection and analysis, decision to publish, or preparation of the manuscript?<br><br>Did you receive funding for this work?"	This work was supported by the Natural Science Foundation of China: "Gap Measurement, Leading Mechanism, and Innovation Leap Research of New Science and Technology Revolution Pilot Technology" (No. 71974041)
Please select the country of your main research funder (please select carefully as in some cases this is used in fee calculation).	CHINA - CN

Powered by Editorial Manager® and ProduXion Manager® from Aries Systems Corporation

as follow-up to "**Financial Disclosure**<br><br>Enter a financial disclosure statement that describes the sources of funding for the work included in this submission. Review the submission guidelines for detailed requirements. View published research articles from *PLOS ONE* for specific examples.<br><br>This statement is required for submission and **will appear in the published article** if the submission is accepted. Please make sure it is accurate.<br><br>**Funded studies**<br>Enter a statement with the following details:<br>• Initials of the authors who received each award<br>• Grant numbers awarded to each author<br>• The full name of each funder<br>• URL of each funder website<br>• Did the sponsors or funders play any role in the study design, data collection and analysis, decision to publish, or preparation of the manuscript?<br><br>Did you receive funding for this work?"	
**Competing Interests**<br><br>Use the instructions below to enter a competing interest statement for this submission. On behalf of all authors, disclose any competing interests that could be perceived to bias this work—acknowledging all financial support and any other relevant financial or non-financial competing interests.<br><br>This statement is required for submission and **will appear in the published article** if the submission is accepted. Please make sure it is accurate and that any funding sources listed in your Funding Information later in the submission form are also declared in your Financial Disclosure statement.	The authors have declared that no competing interests exist.

Powered by Editorial Manager® and ProduXion Manager® from Aries Systems Corporation

View published research articles from *PLOS ONE* for specific examples.<br><br>**NO authors have competing interests**<br><br>Enter: *The authors have declared that no competing interests exist.*<br><br>**Authors with competing interests**<br><br>Enter competing interest details beginning with this statement:<br><br>*I have read the journal's policy and the authors of this manuscript have the following competing interests: [insert competing interests here]*<br><br>* typeset	
**Ethics Statement**<br><br>Enter an ethics statement for this submission. This statement is required if the study involved:<br><br>• Human participants<br>• Human specimens or tissue<br>• Vertebrate animals or cephalopods<br>• Vertebrate embryos or tissues<br>• Field research<br><br>Write "N/A" if the submission does not require an ethics statement.<br><br>General guidance is provided below. Consult the submission guidelines for detailed instructions. **Make sure that all information entered here is included in the Methods section of the manuscript.**	N/A

**Format for specific study types**<br><br>**Human Subject Research (involving human participants and/or tissue)**<br>• Give the name of the institutional review board or ethics committee that approved the study<br>• Include the approval number and/or a statement indicating approval of this research<br>• Indicate the form of consent obtained (written/oral) or the reason that consent was not obtained (e.g. the data were analyzed anonymously)<br><br>**Animal Research (involving vertebrate animals, embryos or tissues)**<br>• Provide the name of the Institutional Animal Care and Use Committee (IACUC) or other relevant ethics board that reviewed the study protocol, and indicate whether they approved this research or granted a formal waiver of ethical approval<br>• Include an approval number if one was obtained<br>• If the study involved *non-human primates*, add *additional details* about animal welfare and steps taken to ameliorate suffering<br>• If anesthesia, euthanasia, or any kind of animal sacrifice is part of the study, include briefly which substances and/or methods were applied<br><br>**Field Research**<br><br>Include the following details if this study involves the collection of plant, animal, or other materials from a natural setting:<br>• Field permit number<br>• Name of the institution or relevant body that granted permission	
**Data Availability**<br><br>Authors are required to make all data underlying the findings described fully available, without restriction, and from the time of publication. PLOS allows rare exceptions to address legal and ethical concerns. See the PLOS Data Policy and FAQ for detailed information.	Yes - all data are fully available without restriction

A Data Availability Statement describing where the data can be found is required at submission. Your answers to this question constitute the Data Availability Statement and **will be published in the article**, if accepted.<br><br>**Important:** Stating 'data available on request from the author' is not sufficient. If your data are only available upon request, select 'No' for the first question and explain your exceptional situation in the text box.<br><br>Do the authors confirm that all data underlying the findings described in their manuscript are fully available without restriction?	
**Describe where the data may be found in full sentences. If you are copying our sample text, replace any instances of XXX with the appropriate details.**<br><br>• If the data are **held or will be held in a public repository**, include URLs, accession numbers or DOIs. If this information will only be available after acceptance, indicate this by ticking the box below. For example: *All XXX files are available from the XXX database (accession number(s) XXX, XXX.*).<br>• If the data are all contained **within the manuscript and/or Supporting Information files**, enter the following: *All relevant data are within the manuscript and its Supporting Information files.*<br>• If neither of these applies but you are able to provide **details of access elsewhere**, with or without limitations, please do so. For example:<br><br>*Data cannot be shared publicly because of [XXX]. Data are available from the XXX Institutional Data Access / Ethics Committee (contact via XXX) for researchers who meet the criteria for access to confidential data.*<br><br>*The data underlying the results presented in the study are available from (include the name of the third party*	All files are available from the National Statistical Yearbook of China (https://www.stats.gov.cn/sj/ndsj/).

and contact information or URL).<br>• This text is appropriate if the data are owned by a third party and authors do not have permission to share the data.<br><br>* typeset	
Additional data availability information:	

Manuscript Click here to access/download;Manuscript;Manuscript.docx

The impact of digital economy on the upgrading of manufacturing structure

Empirical evidence from the Pearl River Delta, China

Ting chen[1], Songlan Zhou[2]

[1] School of Economics and Trade, Guangzhou Huashang College, Zengcheng District, Guangzhou City, Guangdong Province 510000, China; Corresponding: 2652570568@qq.com
[2] School of Econmics and Statistics, Guangzhou University, Guangzhou City, Guangdong Province 510000, China;

Abstract

The global economic situation is in a downturn, and the upgrading of manufacturing structure is a necessary transformation path for the manufacturing industry to achieve high-speed and stable development. The article analyzes the theoretical mechanism of the digital economy affecting the upgrading of manufacturing structure through the endogenous economic growth model, constructs a three-stage mediation effect model, and empirically researches the path of the digital economy affecting the upgrading of manufacturing structure in the Pearl River Delta. The study finds that the digital economy has a significant positive promoting effect on the upgrading of manufacturing structure. In terms of the influencing mechanism, the enhancement of the level of independent innovation and the advancement of the human capital structure are the important paths of the digital economy in promoting the upgrading of manufacturing structure. in addition, the digital economy is negatively correlated with the import of technology, and the import of technology is not an important path for the digital economy to promote the upgrading of manufacturing structure. The research results also find that government support is indispensable to the upgrading of manufacturing structure, and government deployment and control are conducive to accelerating the process of the upgrading of manufacturing structure.

Keywords: Digital economy, The upgrading of manufacturing structure, Endogenous economic growth model, Mediating effect model

Introduction

In recent years, the COVID-19 has broken out, and the uncertainty of the international situation has increased. China's manufacturing industry is facing dual pressures from home and abroad. For external reasons, trade frictions and trade protection have intensified. And there is still a large gap between the level of high-tech and that of developed countries. For internal reasons, the demographic dividends have gradually disappeared, supply side structural reform and core technology constraints. Those all restrict China's transformation from a "big country" to a "strong country" in manufacturing. Under these factors, the proportion of added value of China's manufacturing industry to GDP has continued to decline. Compared to 2006, the proportion of added value of China's manufacturing industry to GDP has decreased from about 32% to about 26% in 2020. Compared with the previous year, the growth rate of added value of the manufacturing industry is less than 0.9%. In the "Made in China 2025" issued by the State Council in 2015, it was pointed out that China's manufacturing industry is still large but not strong, and there is a significant gap between China and a manufacturing powerhouse in terms of independent innovation ability, resource utilization efficiency, industrial structure level, informatization level, etc. The upgrading of the manufacturing industry structure is urgent. In this context, the digital economy, with its high integration and strong penetration, permeates various industries and is accelerating the transformation of traditional industries to digital industries in China.

The digital economy, with digital technology as its core, provides a core driving force for the advancement of manufacturing structure. The 20th National Congress of the Communist Party of China clearly pointed out that China should accelerate the development of the digital economy, strengthen the integration of the digital economy and industry, and create a digital industry with international competitiveness. Vigorously developing an advanced manufacturing system, including high-tech manufacturing and high-end equipment manufacturing, is the main direction for upgrading the industrial structure of China's manufacturing industry, and it has great development potential and space[1]. Therefore, promoting the integration of manufacturing and digital economy, promoting the upgrading of manufacturing structure, is of great significance for the high-quality development of manufacturing.

Literature Review

The upgrading of manufacturing structure

Scholars mainly focus on industrial integration, human capital, environmental regulation, government support and technological progress to study the relationship with the upgrading of manufacturing structure. Xue-Jun L et al (2016)[2] argued that the rise of informatization industry and Internet+ provides a new path for industrial structure upgrading, and the integration of information technology and manufacturing industry is conducive to the upgrading of manufacturing structure. Chengkun Liu (2021)[3] tested through the spatial effect that the quality of human capital is positively correlated with the upgrading of manufacturing structure, and human capital has a positive impact on industrial structure upgrading[4]. Porter et al. (1995)[5] hypothesis that, although environmental regulations in the short term will lead to an increase in enterprise costs, but in the long term, enterprises will avoid the negative impact of environmental regulations by strengthening technological innovation and management, which indirectly promotes the upgrading of industrial structure. Xiqiang Chen and Yuanhai Fu (2017)[6] indicate that the government tends to form an administrative monopoly, industrial policy distortion, local protection, market segmentation and so on, leading to resource mismatch, is not conducive to the upgrading of manufacturing structure. However, Xiangsong Ye and Jing Liu (2020)[7] believes that government support strongly promotes the progress of high-end manufacturing science and technology level, and technological progress is conducive to the upgrading of manufacturing structure[8]. Jingrong Dong and Wenqing Zhang (2019)[9]classified the sources of technological progress into four kinds of technological imports, foreign investment, cooperative research and development(R & D), and independent R & D, and estimated them through the method of SUR, analyzing their relationship with the upgrading of manufacturing structure, and the study found that technology import, cooperative R&D as the source of technology has a significant positive promotion effect on the upgrading of manufacturing structure in China, but for the technology-intensive eastern region, the upgrading of manufacturing structure is mainly affected by foreign direct investment, independent R&D.

Digital economy and the upgrading of manufacturing structure

The digital economy represented by Big data, artificial intelligence, Internet plus and information technology has realized the deep integration of digital technology and the real economy, promoting the accelerated transformation of traditional manufacturing industry. From the perspective of factor allocation, the digital economy realizes information transparency through Big data, reduces manufacturing production costs, optimizes

resource allocation, drives innovative development, improves manufacturing enterprise production efficiency[10], and further promotes the upgrading of manufacturing structure. From the perspective of impact approach, Yong Zhou et al. (2022)[11] found that the digital economy has significantly promoted the upgrading of manufacturing structure through the study of the mediating effect model, in which innovation ability and Total factor productivity have a significant partial mediating effect on this promotion. In addition, Yanze Cai et al. (2021) [12]pointed out that the innovation environment, including talent gathering and financial development, plays a moderating role in the digital economy's significant promotion of the upgrading of manufacturing structure, And this regulatory effect has a certain threshold effect. When talent aggregation and financial development reach a certain level, the effect of digital economy on promoting the upgrading of manufacturing structure is significantly enhanced.

Based on existing research, this study has two innovations, first, by using an endogenous economic growth model, the impact of the digital economy on the upgrading of manufacturing structure is analyzed, providing theoretical support for the impact of the digital economy on industrial growth rate. Existing research mostly focuses on empirical analysis of the impact of various economic variables on the upgrading of manufacturing industry structure, but generally lacks an inherent theoretical analysis of the impact of the digital economy on the upgrading of manufacturing industry structure. This article takes into account the characteristics of the digital economy and describes it as "knowledge", which can be a technology that affects labor efficiency or a theory that affects capital operation efficiency. It integrates the Cobb Douglas production function and explores the internal mechanism of the impact of the digital economy on the upgrading of manufacturing structure. Second, Considering the transmission effect of economic variables and the diffusion effect of digital economy, this article uses human capital and technological progress as intermediary variables to analyze the impact path of digital economy on the upgrading of manufacturing structure. On the other hand, the article focuses on the Pearl River Delta, as a frontier area of advanced technology, capital accumulation and economic development, it has a strong reference significance on whether and how the digital economy affects the upgrading of manufacturing structure. It is of great significance to promote the integration of digital economy and manufacturing industry, accelerate the transformation of traditional manufacturing industry in the Pearl River Delta, and break through the bottleneck of manufacturing industry development in the Pearl River Delta.

Research Hypothesis

With the advent of the Internet and big data, data elements, digital technology, and digital economy have emerged. The digital economy, with information and communication technology (ICT) as its core, is the third new economic form brought about by informatization, following agricultural and industrial economies. According to the endogenous economic growth model, technological progress is endogenous, and capital is divided into physical capital and knowledge capital. The former has the characteristic of diminishing returns to scale, while the latter is not. Knowledge investment is the key to high equilibrium growth rates. Knowledge has non exclusivity, and both the capital and human capital departments can use the entire knowledge stock (A). In the endogenous economic growth model, product A produced by research and development is described as knowledge, which exists in many forms, including technologies that improve labor efficiency and theories that improve capital operation efficiency. This characteristic is completely consistent with the characteristics of the digital economy. The digital economy can not only optimize management models through digital technology[13], improve the efficiency of production, exchange, distribution, and consumption, reduce transaction costs, and promote enterprises to achieve economies of scale[14] but also the data elements derived from the digital economy play a central role in production, it improves the synergy between labor, capital, and other factors by utilizing valuable information[15]. Data elements penetrate through various stages of production through high penetration rates, accelerating capital operation, greatly shortening the capital operation cycle, and doubling the efficiency of capital operation. Therefore, this article believes that the digital economy can affect both labor and capital, and lists the following Cobb Douglas production function:

$$Y(t) = [(1-a_k)A(\mathrm{t})\mathrm{K}(\mathrm{t})]^{\alpha}[(1-a_L)A(\mathrm{t})L(\mathrm{t})]^{1-\alpha}, \quad 0<\alpha<1 \qquad (1)$$

Calculate：

$$Y(t) = A(\mathrm{t})[(1-a_k)\mathrm{K}(\mathrm{t})]^{\alpha}[(1-a_L)L(\mathrm{t})]^{1-\alpha}, \qquad 0<\alpha<1 \qquad (2)$$

Neglecting depreciation of capital, the change in capital is

$$\dot{K}(\mathrm{t}) = sY(t) = s(1-a_k)^{\alpha}(1-a_L)^{1-\alpha}A(\mathrm{t})K(\mathrm{t})^{\alpha}L(\mathrm{t})^{1-\alpha}, \quad 0<\alpha<1 \qquad (3)$$

If $c = s(1-a_k)^{\alpha}(1-a_L)^{1-\alpha}$

$$g_{k=}\frac{K(\dot{t})}{K(t)} = cA(\mathrm{t})[\frac{L(\mathrm{t})}{K(\mathrm{t})}]^{1-\alpha} \qquad (4)$$

Taking logarithmic derivative over time yields：

$$\frac{\dot{g}_k}{g_k} = g_A + (1-\alpha)(n-g_k) \qquad (5)$$

When $\dot{g_k}$=0

$$g_k = \frac{g_A}{1-\alpha} + n \tag{6}$$

Y, K and L respectively represent output, capital, and labor, a_k is the proportion of capital invested in research and development, and $(1 - a_k)$ is the proportion of capital invested in production. a_L is the proportion of research and development investment in labor. $(1 - a_L)$ is the proportion of labor input into production. g_A is the growth rate of knowledge. g_k is the growth rate of capital, and n is the growth rate of labor. The production of new knowledge depends on the capital, labor, and technological level of research, thus obtaining the following equation:

$$\dot{A(t)} = \mathrm{B}[a_k\mathrm{K(t)}]^{\beta}[a_L L(\mathrm{t})]^{\gamma}\mathrm{A(t)}^{\theta} \quad ,\mathrm{B}>0, \beta \geq 0, \gamma \geq 0 \tag{7}$$

$$g_A = \frac{\dot{A(t)}}{A(t)} = \mathrm{B}[a_k\mathrm{K(t)}]^{\beta}[a_L L(\mathrm{t})]^{\gamma}\mathrm{A(t)}^{\theta-1} \tag{8}$$

Taking logarithmic derivative over time yields：

$$\frac{\dot{g_A}}{g_A} = (\theta - 1)g_A + \beta g_k + \gamma n \tag{9}$$

When $\dot{g_A}$=0

$$g_k = \frac{1-\theta}{\beta} g_A - \frac{\gamma n}{\beta} \tag{10}$$

B is the conversion parameter, θ is the impact of knowledge stock on R&D rate. If θ is greater than 1, it indicates that the knowledge stock has a huge impact on the production of new knowledge. The marginal increase in the level of knowledge stock will generate a large amount of new knowledge, leading to a continuous increase in the growth rate of knowledge. If θ Equal to 1, the increase in knowledge stock will be proportional to the addition of new knowledge. If θ is less than 1, it indicates that the increase in knowledge stock has a limited effect on new knowledge and will gradually converge. According to the characteristics of the digital economy, as a new production factor, the digital economy integrates with various industries, eliminates industrial barriers, avoids adverse selection and external diseconomy caused by information asymmetry in industries, effectively saves enterprise operating costs and menu costs, improves industrial allocation efficiency, and forms Pareto optimality. Those have a huge impact on the increase of new knowledge. Therefore, this article assumes that in the context of the digital economy, θ＞ 1. Because β≥ 0, θ+ β＞ 1. Draw the following coordinate graph according to equations (6) and (10), as shown in Figure 1, with the two curves gradually separating.

Figure 1 Capital and Knowledge Growth Rate Curve

The initial values of model parameters and knowledge (A), capital (K), and labor (L) determine the initial value of g_A, g_k. According to Figure 1, in the context of the digital economy, no matter where the initial value of g_A, g_k is, it will enter the middle region between the curve $\dot{g}_k$=0 and $\dot{g}_A$=0, and $\dot{g}_k$, $\dot{g}_A$ will be greater than 0. In other words, g_k, g_A will continue to grow, and the growth rate of capital and knowledge will continue to increase. According to the production function equation (2), the output growth rate can be written as:

$$\frac{\dot{Y}(t)}{Y(t)} = g_Y = g_A + \alpha g_k + (1-\alpha)n \tag{11}$$

According to equation (11), in the context of the digital economy, as the growth rate of knowledge and capital continues to increase, the output growth rate also continues to increase. What's more, the Kuznets rule points out that within the manufacturing industry, the fastest-growing sectors are emerging industries closely related to modern technology. Based on the analysis above, this article believes that the digital economy can not only comprehensively promote the increase of manufacturing output, but also have a more significant promoting effect on high-end technology sectors. Therefore, the following hypothesis is proposed.

Hypothesis 1: The digital economy can promote the upgrading of manufacturing structure.

The theory of endogenous economic growth points out that technological progress and the accumulation of human capital are the fundamental driving forces for sustained economic growth. Accelerating the development of the digital economy helps to promote technological progress and the accumulation of human capital, thereby assisting in the structural adjustment of the manufacturing industry, which is of great significance for promoting high-quality development of the manufacturing industry.

The development of the digital economy has accelerated the progress of industrial digitization and digital industry, improved capital allocation and utilization efficiency, provided sufficient funds for R & D innovation, stimulated innovation vitality, and thus promoted technical progress[16]. From the supply side perspective, technical progress injects new momentum into the manufacturing industry dominated by information technology, improves enterprise production efficiency, changes traditional production methods, reduces production costs, increases producer surplus, and promotes the upgrading of manufacturing structure. From the demand side, technical progress can create more diverse and diverse goods for consumers, provide more convenient and efficient services, enhance consumer experience, and drive consumption. The increase in consumer demand will

inevitably promote technical progress, thereby promoting the upgrading of manufacturing structure. The new economic growth theory points out that technical progress is conducive to improving the core competitiveness of industries and achieving a leap from low to high added value in industries[17]. Therefore, this article proposes a second hypothesis:

Hypothesis 2: The digital economy promotes the upgrading of manufacturing structure by promoting technology progress

The digital economy is the product of the information age. With the development of digital technologies such as artificial intelligence and Big data, traditional human capital can no longer meet the requirements of digital technology. The improvement of digital technology cannot be separated from the accumulation of scientific knowledge and technological innovation ability of high digital literacy talents[18]. On the one hand, in order to enhance their own development space, win better working conditions, and obtain more job opportunities, workers continuously improve their professional knowledge and skills through education and training to meet the needs of the times[19]. The digital economy has promoted the advancement of human capital structure through the effects of expansion, deepening, and career creation[20]. On the other hand, according to the theory of human capital, the accumulation and upgrading of human capital structure are the third fundamental change that occurs in productivity. Human capital has the increasing effect of returns to scale, which is conducive to the increase of income from other input factors. And it is the basis of industrial structure change, and also determines the direction, speed and effect of industrial structure change. Human capital structure plays an important role in industrial structure transformation by influencing production efficiency, innovation performance, and agglomeration effect[21]. Therefore, this article proposes a third hypothesis:

Hypothesis 3: The digital economy promotes the advancement of manufacturing structure through the advancement of human capital structure.

Based on the analysis above, the relationship between the digital economy and the upgrading of manufacturing structure is shown in Figure 2.

Figure 2: The Transmission Path of the Impact of Digital Economy on the Upgrading of Manufacturing Structure

Method and data

Benchmark mode

Based on the mechanism analysis of the impact of the digital economy on the upgrading of manufacturing structure mentioned above, in order to test the research hypothesis, the following benchmark regression model is constructed for the direct transmission mechanism of the impact of the digital economy on the upgrading of manufacturing structure:

$$\text{ManuH}_{it} = \alpha_0 + \alpha_1 Digital_{it} + \sum_k \alpha_2 \, Control + \delta_i + \varepsilon_i + \mu_{it} \qquad (12)$$

Among them, i and t represent the sample individuals and time, respectively. ManuH represents the upgrading of manufacturing structure. Digital is the level of digital economy development calculated based on principal component analysis method. Control is the control variable. $\delta_i$and ε_i represents individual and time effects, respectively, μ_{it} is a random interference term.

Mediated effect model

In addition to the direct effect reflected in equation (12) above, in order to discuss the possible transmission mechanism of the impact of the digital economy on the upgrading of manufacturing structure, we test whether independent innovation, the import of technology, and human capital are intermediary variables between the two. The specific testing steps are as follows: On the basis of the significant passing of the coefficients in the linear regression model (12) of the digital economy on the upgrading of manufacturing structure, and then construct linear regression models (13), of the digital economy on intermediary variables and regression equations (14) of the impact of the digital economy and intermediary variable on the upgrading of manufacturing structure:

$$\text{ME}_{\text{it}} = \beta_0 + \beta_1 \text{Digital}_{\text{it}} + \sum_{\text{k}} \beta_2 \, \text{Control} + \delta_{\text{i}} + \varepsilon_{\text{i}} + \mu_{\text{it}} \qquad (13)$$

$$\text{ManuH}_{\text{it}} = \gamma_0 + \gamma_1 \text{Digital}_{\text{it}} + \gamma_2 \text{ME}_{\text{it}} + \sum_{\text{k}} \gamma_3 \, \text{Control} + \delta_{\text{i}} + \varepsilon_{\text{i}} + \mu_{\text{it}} \qquad (14)$$

ME is mediating variable. According to the mediated effect model analysis steps of Zhonglin Wen and Lei Zhang (2004)[22], judge whether mediating effect exists and the type of mediating effect. The judgment steps are shown in figure 3.

Figure 3：Diagram of mediating effect

Data sources

Considering the availability of data, this article uses data from nine cities in the Pearl River Delta from 2012 to 2021, all of which are sourced from the Guangdong Statistical Yearbook.

Dependent variable

The industrial structure upgrading of the manufacturing industry mainly involves the transformation and upgrading from resource and labor-intensive manufacturing to technology and capital intensive manufacturing, and from traditional low-end manufacturing to modern, advanced, and emerging manufacturing[1]. This article refers to the OECD's classification method for manufacturing industry and divides it into low-end manufacturing, mid-end manufacturing, and high-end manufacturing[1], and the study refer to the approach of Zhanxiang, F et al.(2016)[23], adopt the proportion of high-end manufacturing output value to the total manufacturing output value to measure the degree of the upgrading of manufacturing structure. The larger the proportion, the higher the degree of the upgrading of manufacturing structure.

Independent variable

This article, from the perspective of digital application and output, selects five indicators, including internet penetration rate, mobile phone penetration rate, fixed line penetration rate, digital output, and digital technology related practitioners, and uses principal component analysis to synthesize a digital economy development index to illustrate the level of regional digital economy development.

Mediating variable

Technical progress is the fundamental way to optimize the structure of the manufacturing industry[24]. The article divides technical progress into two aspects: independent innovation and the import of technology. Scholars mainly measure independent innovation in terms of income and expenditure. This article refers to Jie Zhang et al. (2020)[25], who use per capita scientific and technological activity expenditure to measure the level of independent innovation in terms of expenditure. Considering the impact of the import of technology on domestic

---

1. 1 Low-end manufacturing: food processing and manufacturing, beverages, tobacco, textiles, clothing, leather, wood, furniture, paper making, printing and sports goods, and other manufacturing industries.
2. Middle-end manufacturing: petroleum processing, coking and nuclear pigment processing, rubber and plastics, non-metallic minerals, Ferrous smelting, non-ferrous metal smelting and metal products.
3. High-end manufacturing: chemical medicine, general equipment, specialized equipment, transportation equipment, electrical machinery and equipment, computer communication and electronic equipment, instruments and meters, etc.

enterprises, foreign investment participation is adopted that the proportion of total output value of foreign enterprises to total industrial output value to measure the level of the import of technology.

Advanced human capital structure: The level of human capital can improve the labor efficiency of the manufacturing industry and promote the upgrading of manufacturing structure. This article considers that the digital economy requires a high level of talent literacy, and the number of ordinary undergraduate graduates has not yet reflected the human capital level of digital technology talents. Therefore, this article uses the number of scientific and technological personnel to measure the advancement of human capital structure.

Control variable

This article selects the per capita GDP level to measure the economic development level of a region. Using the proportion of non-state-owned industrial output value to total industrial output value to measure the degree of marketization. The proportion of fiscal expenditure to GDP indicates government intervention. The ratio of total import and export volume to GDP measures the level of opening-up. The ratio of year-end urban population to year-end permanent population measures the urbanization rate. The variable description and descriptive statistics are shown in Table 1.

Table 1 Variable indicators and descriptive analysis

Variable Type	Variables	Variable Symbols	Mean	Std. Dev.	Min	Max
Dependent variables	the upgrading of manufacturing structure	ManuH	0.596	0.165	0.261	0.848
Independent variable	Digital economy	Digital	1.67e-08	0.977	-1.825	2.544
Mediating variable	The import of technology	Tech-int	0.458	0.116	0.216	0.713
	Independent innovation	Tech-inv	94.131	178.173	2.111	769.221
	Advanced human capital structure	High-hum	2 123.99	4 705.67	58	191 94
Control variable	Economic development level	PGDP	9.018	3.594	3.240	17.366
	Marketization	Mark	0.861	0.097	0.556	0.959
	Government intervention	Gov	0.125	0.039	0.018	0.203

	Level of opening-up	Open	0.864	0.517	0.153	2.284
	Urbanization	Urban	0.805	0.164	0.426	1

Empirical results

Baseline analysis

This article uses a stepwise regression method to analyze the relationship between the digital economy and the upgrading of manufacturing structure by sequentially adding government intervention, urbanization, marketization and Level of opening-up in model (12), Table 2. According to the benchmark regression results in Table 2, the research results strongly indicate that the digital economy has a significant positive impact on the upgrading of manufacturing structure. For every 1 unit increase in the development level of the digital economy, the upgrading of manufacturing structure increases by 0.0227 units. The digital economy, through its highly technological characteristics, is conducive to stimulating the vitality of regional independent innovation, improving the level of independent innovation, promoting the development of industrial technology and the integration of technology and industry, promoting the generation of economies of scale and scope in industries, greatly reducing production costs, improving industrial efficiency, and promoting the upgrading of manufacturing structure, confirming the hypothesis 1.

According to (2) to (6) in Table 2, the impact of control variables on the upgrading of manufacturing structure is analyzed. The results show that government intervention is conducive to the upgrading of manufacturing structure in the Pearl River Delta region, and the impact is very significant. For every unit increase in government intervention, the upgrading of manufacturing structure increases by 0.45 units. In addition, the improvement of urbanization level and the degree of marketization are not conducive to the upgrading of manufacturing structure in the Pearl River Delta region. Due to the fact that urbanization is the result of population migration under factors such as production and consumption structure, income situation, and government expenditure distribution[26], urbanization can promote the transformation of employment structure and promote urban industrialization. Although urbanization can bring more labor and investment, it mainly targets low-end industries and has a significant crowding out effect on high-tech industries[27], which is not conducive to the upgrading of manufacturing structure. Furthermore, the improvement of marketization degree means that the government's control over the market economy is gradually relaxed, and the economy is automatically regulated by the market. Analysis shows that the upgrading of manufacturing structure in the Pearl River Delta region is undergoing a

transformation with the development of the digital economy. The digital economy in the Pearl River Delta region is still in a stage of rapid development but the development level is not yet fully mature and uneven. The technological R & D, capital investment, and human development required for the upgrading of manufacturing structure still require strong government support. The improvement of marketization has a significant inhibitory effect on the technological level of high-end manufacturing industry, which is not conducive to the upgrading of manufacturing structure in the Pearl River Delta at present. According to the benchmark regression results in Table 2, it can also be found that level of opening-up has a negative impact on the upgrading of manufacturing structure in the Pearl River Delta, but this effect is not significant. Therefore, it can be seen that the increase in the degree of opening up of the Pearl River Delta to the outside world is not conducive to the upgrading of manufacturing structure in the Pearl River Delta.

Table 2 Regression results

						Robustness test results	
Variables	（1）	（2）	（3）	（4）	（5）	(6)	(7)
	ManuH	ManuH	ManuH	ManuH	ManuH	ManuH	Manuhigh
Digital	0.018 8*** (4.06)	0.015 1*** (3.20)	0.022 7*** (4.16)	0.023 0*** (4.42)	0.022 7*** (3.45)		0.02 299*** (3.09)
L.digital						0.022 9* ** (2.94)	
Gov		0.296 8*** (2.46)	0.367 3*** (3.06)	0.455 4*** (3.85)	0.449 4*** (3.16)	0.339 3 ** (2.37)	0.630 1*** (3.93)
Urban			-0.456 2** (-2.55)	-0.492 8*** (-2.88)	-0.490 7*** (-2.81)	-0.530 5 *** (-2.80)	-0.441 4** (-2.24)
Mark				-0.175 8*** (-2.93)	-0.177 8*** (-2.70)	-0.218 6 *** (-3.44)	-0.350 9*** (-4.72)
Open					-0.001 2 (0.939)	-0.006 4 (-0.38)	-0.002 5 (-0.14)
Constant	0.596 0*** (242.50)	0.558 9*** (36.56)	0.917 3*** (6.49)	1.087 1*** (7.41)	1.089 0*** (7.28)	1.179 4 *** (7.03)	1.079 3*** (6.39)

Observations	90	90	90	90	90	81	90
Fixed effect	Yes	Yes	Yes	Yes	Yes	Yes	Yes
R-square	0.622 2	0.423 4	0.300 7	0.360 5	0.360 5	0.317 0	0.448 5

Notes: ***, **, and * in the table denote significance levels at 1%, 5%, and 10%, respectively, and standard deviations are in parentheses, as below

The analysis of mediating effect model

According to (8), (10), and (12) in Table 3, it shows that the digital economy has a significant impact on the level of independent innovation, the import of technology, and the upgrading of human capital structure. Among them, the development of the digital economy has a significant positive impact on the level of independent innovation and the upgrading of human capital structure. Due to the high-tech nature of the digital economy, the development of the digital economy provides broader development space and innovative vitality for independent innovation, thereby promoting the improvement of the level of independent innovation. The development of the digital economy cannot be separated from the demand for high-skilled personnel, and with the increase in demand, the number of high-skilled personnel also increases, therefore, the development of the digital economy is conducive to the promotion of the advanced structure of human capital. In addition, the study found that the development of the digital economy in the Pearl River Delta is negatively correlated with the import of technology. On the one hand, because the import of technology not only requires great capital investment, but also inhibits the vigor of innovation, resulting in the negative impact of the import of technology is greater than the positive impact of the technological spillovers generated by the import of technology, on the other hand, with the development of the digital economy, the domestic information technology has been greatly improved, due to the technological barrier, the marginal utility of the import of technology has decreased substantially, and the marginal reward brought by independent innovation is much larger than the marginal reward brought by the import of technology. With the development of digital economy, the requirements for the level of digital technology are getting higher and higher, and the development of the domestic digital technology is getting more and more mature, and no longer relies on the import of technology. If the digital economy wants to realize the long-term and stable development, it is necessary to grasp the technology in its own hands.

This paper uses the three-step's the mediating effect model, introduces the level of independent innovation, the import of technology, and human capital as intermediary variables, and analyzes the path of digital economy affecting the upgrading of manufacturing structure in the Pearl River Delta. According to (9) in Table 3, after

adding the level of independent innovation, the coefficient of the digital economy is 0.019, which is less than the result of (5) in Table 2, which is 0.023, and the result is significant. Furthermore, the coefficient of independent innovation level is positive, but the result is not significant. According to the test steps of the mediating effect model in figure 2, the Bootstrap test was carried out, and the confidence interval was [-0.046, -0.012], excluding zero, indicating that there was a partial mediating effect, and the mediation effect accounted for 17.5%. The level of independent innovation is the transmission path for the digital economy to promote the upgrading of manufacturing structure in the Pearl River Delta. According to (11) in Table 3, after the import of technology, the coefficient of the digital economy is 0.007, the result is less than 0.023, but the result is not significant. What's more, the coefficient of the import of technology is significantly negative, and bootstrap test shows that the confidence interval is [-0.011, 0.013], including zero, thereby hypothesis 2 is not entirely correct, independent innovation has intermediary effect, but the import of technology does not. The import of technology is not an intermediary variable for the digital economy to promote the upgrading of manufacturing structure in the Pearl River Delta. According to (13) in Table 3, when the intermediary variable of human capital structure upgrading is added, the coefficient of digital economy is 0.019, less than 0.023, and the result is significant. The result of human capital structure upgrading is positive, but not significant. Bootstrap analysis is conducted, and the confidence interval is [-0.027, -0.001], excluding zero, it indicates that there is a partial mediating effect, and the mediation effect is calculated to account for 17.4%, which confirms that the previous hypothesis 3 is correct, therefore, the digital economy can promote the upgrading of manufacturing structure in the Pearl River Delta region by promoting the upgrading of human capital structure.

Table 3 The analysis results of mediating Effect Model

Variables	（8）	（9）	（10）	（11）	（12）	（13）
	Tech-inv	ManuH	Tech-int	ManuH	Highhum	ManuH
Digital	69.754***<br>(4.23)	0.0190***<br>(3.30)	-<br>0.085***<br>(-8.17)	0.007<br>（1.00 ）	388.185*<br>(1.73)	0.019***<br>（2.94）
Tech-inv		3.72e-06<br>（ 0.08）				
Tech-int				-0.296***<br>(-5.67)		

Highhum						3.46e-06<br>(1.07)
Constant	628.391*<br>(1.68)	0.696***<br>(12.56)	0.823***<br>(2.80)	1.159***<br>（9.17）	19 724.59***<br>(4.74)	0.873***<br>(5.94)
Fixed effect	Yes	Yes	Yes	Yes	Yes	Yes
Observation	90	90	90	90	90	90
R-square	0.408	0.535	0.717	0.529	0.570	0.299
Bootstrap analysis 95% confidence interval		[-0.046, -0.012]		[-0.011,0.013]		[-0.027,-0.001]
Total effect (α_1)	0.023				0.023	
Direct effect (γ_1)	0.018 97				0.019 0	
Mediation effect ($\beta_1\gamma_2$)	0.004 03				0.004	
Results	Partial mediation effect（17.5%）		No mediation effect		Partial mediation effect（17.4%）	

Notes: ***, **, and * in the table denote significance levels at 1%, 5%, and 10%, respectively, and standard deviations are in parentheses, as below

Robustness test

This article adopts the method of stepwise regression and sequentially adds variables for regression. The regression results are shown in Tables 2 (1) - (5), and the coefficients and significance of the variables clearly indicate robustness. In addition, considering that the digital economy may have a certain time lag effect, this article uses the digital economy to replace the digital economy variable with a lag period for re regression. The results are shown in Table 2, Model (6), and the positive and negative directions and significance of the variable coefficients are completely the same, indicating robustness. Considering the measurement of the dependent variable, referring to Donghua Yu and Kun Zhang (2020)[28], the manufacturing industry is divided into labor

intensive manufacturing, capital intensive manufacturing, and technology intensive manufacturing[1]. The index of the upgrading of manufacturing structure is re measured (Manuhigh) using the ratio of technology intensive manufacturing output value to total manufacturing output value, and a robustness test is conducted. The results are shown in Table 2, Model (7) that the model coefficients and significance are basically consistent with the benchmark regression results, and it confirms the robustness of the results once again.

Table 4 tests the robustness of the mediating effect model. It refers to the above practice, replaces the measurement indicators of the upgrading of manufacturing structure, and conducts regression and test again. The results are shown in Table 4, which is basically consistent with the results in Table 3. Therefore, the results are robust

Table 4 The robustness test results of the mediating effect model.

Variables	Manuhigh	Manuhigh	Manuhigh
Digital	0.021**<br>(2.51)	-0.000 6<br>（-0.09）	0.019**<br>（2.27）
Tech-inv	0.000 031<br>(0.60)		
Tech-int		-0.360***<br>（-6.71）	
Highhum			4.47e-06<br>（1.10）
Constant	1.060***	1.332***	0.977***

4. [1] **Labor intensive manufacturing industry:** agricultural and sideline Food processing, food manufacturing industry, rubber and plastic products industry, metal products industry, textile industry, textile clothing and clothing industry, leather, fur, feather and its products and shoemaking industry, wood processing and wood, bamboo, rattan, palm, grass products industry, furniture manufacturing industry, non-metallic mineral products industry, printing and recording media reproduction industry, cultural and educational, arts and crafts, sports and entertainment products manufacturing industry.
5. **Capital intensive manufacturing industry:** petroleum processing, coking and nuclear fuel processing industry, Ferrous metal smelting and rolling processing industry, nonferrous metal smelting and rolling processing industry, chemical raw materials and chemical products manufacturing industry, chemical fiber manufacturing industry, general equipment manufacturing industry, wine, beverage and refined tea manufacturing industry, tobacco products industry, paper making and paper products industry.
6. **Technology intensive manufacturing industry:** specialized equipment manufacturing, automobile manufacturing, railway, shipbuilding, aerospace and other transportation equipment manufacturing, electrical machinery and equipment manufacturing, instrument and meter manufacturing, pharmaceutical manufacturing, computer, communication and other electronic equipment manufacturing.

	（6.14）	（9.54）	（5.08）
Fixed effect	Yes	Yes	Yes
Observation	90	90	90
R-square	0.451	0.656	0.457
Bootstrap analysis 95% confidence interval	[-0.048,-0.014]	[-0.015 ,0.011]	[-0.038, -0.005]
Total effect (α_1)	0.023		0.023
direct effect (γ_1)	0.021		0.019
Mediation effect ($\beta_1\gamma_2$)	0.002		0.004
Results	Partial mediating effect 9.6%	No Mediating effect	Partial mediating effect 17.8%

Notes: ***, **, and * in the table denote significance levels at 1%, 5%, and 10%, respectively, and standard deviations are in parentheses, as below

Conclusion and political implication

With the advent of the information age, the integration of digital economy and manufacturing has become the main way to promote the upgrading of manufacturing structure. The digital economy through digital technology can improve resource allocation efficiency, save production costs, and optimize industrial structure. The development of digital economy has a significant role in promoting the upgrading of manufacturing structure in the Pearl River Delta, and this role has a certain mediation effect. The digital economy mainly promotes the upgrading of manufacturing structure in the Pearl River Delta by improving the level of independent innovation and promoting the upgrading of the human capital structure. The mediation effect of the level of independent innovation is greater than the mediation effect of the upgrading of the human capital structure. In addition, the development of the digital economy is conducive to the improvement of the level of independent innovation and the advanced structure of human capital, but the import of technology is negatively correlated with the digital economy. The import of technology is not a way for the digital economy to promote the upgrading of manufacturing structure, and the import of technology is not conducive to the upgrading of manufacturing structure in the Pearl River Delta. Furthermore, the research results also indicate that the progress of the upgrading of manufacturing structure in the Pearl River Delta is still in a steady improvement stage, and the technology and

resource allocation are still not fully mature, technology R & D, industrial integration still require government support and regulation, and government intervention has a significant positive impact on the upgrading of manufacturing structure in the Pearl River Delta. In addition, the level of marketization and urbanization is not conducive to the upgrading of manufacturing structure in the Pearl River Delta, further confirming the instability of the stage of the upgrading of manufacturing structure in the Pearl River Delta, which cannot do without government intervention.

Based on the empirical results of this study and a real socio-economic environment, this paper has the following policy implications. Firstly, the development of the digital economy has brought new opportunities to the development of the manufacturing industry, and the manufacturing industry want to achieve further development, it is necessary to undergo a transformation of the upgrading of manufacturing structure. Enterprises should strengthen the integration of the digital economy and the manufacturing industry by fully leveraging the high integration characteristics of the digital economy and infiltrating the digital economy into all aspects of the manufacturing process, thereby improving the efficiency of manufacturing resource allocation and achieving economies of scale, which is the key to achieving the upgrading of manufacturing structure for enterprises. Secondly, enterprise should strengthen patent certification and management and enhance awareness of intellectual property rights to provide a good and fair development platform for independent technological innovation. What's more, government should increase investment in independent innovation and encourage technological innovation, cross integration, and application to provide broad development channels for independent innovation. At the same time, government should emphasize the dominant position of technological innovation in enterprises, encourage enterprises to independently research and develop advanced technologies, and reduce dependence on the import of technology, thereby achieve a leap in technological level towards international standards. Enterprise should hold the initiative of technical progress in their own hands and promote the high-speed development of the digital economy through technical progress. Thirdly, government can cultivate professional high-tech innovation and R & D talents by promoting school enterprise cooperation to improve the digital literacy of human capital, and enterprise can improve the efficiency of "learning by doing" through providing vocational and technical training for enterprise talents to promote the advancement of human capital structure and provide talent reserves for the integration of the digital economy and manufacturing industry. Fourthly, government should steadfastly and continuously strengthen support for high-end manufacturing and leverage the advantages of the national system to increase investment in infrastructure construction for the integration of digital economy and manufacturing industry, focus on basic R & D, and use digital technology as

a breakthrough point for the upgrading of manufacturing structure, thereby accelerating the process of the upgrading of manufacturing structure.

Author Contributions: Thanks for the corresponding author for collaborating with me to complete this article including but not limited to formal analysis and data curation. writing—original draft preparation, Ting Chen; writing—review and editing, Songlan Zhou; All authors have read and agreed to the published version of the manuscript.

Funding: This research was funded by the Natural Science Foundation of China: "Gap Measurement, Leading Mechanism, and Innovation Leap Research of New Science and Technology Revolution Pilot Technology", grant number 71974041.

Institutional Review Board Statement: Not applicable.

Informed Consent Statement: Not applicable.

Data Availability Statement: The analysis data used in the article are all from the National Statistical Yearbook of China, and the data results presented in the article are all analyzed using Stata software. The data presented in this study are available on request from the corresponding author

Conflicts of Interest: The authors declare no conflicts of interest.

References

1. Xiao-xiao, G. K.-s. T., Accelerating the Construction of a New Development Pattern and Path of Transformation and Upgrading of Manufacturing. *China Industrial Economics* **2021**, (11), 44-58.http://10.19581/j.cnki.ciejournal.2021.11.005.
2. Xue-Jun, L.; Han, L.; Luo, H.; Nie, H., Technological Embedding, Industrial Integration, Industrial Upgrading----Discuss about the Role of Information Technology in Industrial Upgrading and Transformation. *International Journal of Economics and Finance* **2016,** 8, (2).http://10.5539/ijef.v8n2p39.
3. Chengkun, L., Research on the Spillover Effect of Human Capital on the Manufacturing Structure

Upgrading. *Journal of Technical Economics & Management* **2021**, (12), 108-112

4. Teixeira, A. A. C.; Queirós, A. S. S., Economic growth, human capital and structural change: A dynamic panel data analysis. *Research Policy* **2016,** 45, (8), 1636-1648.http://doi.org/10.1016/j.respol.2016.04.006.

5. C., P. M. E. V. D. L., Toward a new conception of the environment competitiveness relationship. *Journal of Economic Perspectives* **1995,** 9, (4), 97-118

6. Xiqiang Chen, Y. F., Yun Luo., Research on the impact of government led regional economic integration strategy on the structural optimization of manufacturing industry: A case study of the Pan Pearl River Delta region. *China Soft Science* **2017**, (09), 69-81

7. Xiangsong Ye , J. L., The impact of government support and market-orient reforms on manufacturing technology progress *Economic Research Journal* **2020,** 55, (05), 83-98

8. Guangcan Huang, J. W., Lili Ma, Research on t China's manufacturing upgrading from the perspective of global value chains: Based on whole industry chains. *Social Sciences in Guangdong* **2019**, (01), 54-64

9. Jingrong Dong, W. Z., Technology source, technology progress bias and China's manufacturing upgrading --Thoughts on Dual Circulation New Development Pattern *Forum on Science and Technology in China* **2021**, (10), 71-82.http://10.13580/j.cnki.fstc.2021.10.009.

10. Qunhui Huang, Y. Y., Songlin Zhang, Internet development and productivity growth in manufacturing industry: Internal mechanisms and China experience. *China Industrial Economics* **2019**, (08), 5-23.http://10.19581/j.cnki.ciejournal.2019.08.001.

11. Yong Zhou , H. W., Zhao'an Han, The impact of the digital economy on the transformation and upgrading of the manufacturing industry. *Statistics & Decision* **2022,** 38, (20), 122-126.http://10.13546/j.cnki.tjyjc.2022.20.024.

12. Yanze Cai , X. G., Mei Jin, Digital economy, innovation environment and transformation and upgrading of manufacturing industry. *Statistics & Decision* **2021,** 37, (17), 20-24.http://10.13546/j.cnki.tjyjc.2021.17.004.

13. Martínez-Caro, E.; Cegarra-Navarro, J. G.; Alfonso-Ruiz, F. J., Digital technologies and firm performance: The role of digital organisational culture. *Technological Forecasting and Social Change* **2020,** 154, 119962.http://doi.org/10.1016/j.techfore.2020.119962.

14. Peiwen Bai, Y. Z., Digital economy, declining demographic dividends and the rights and interests of low-and-medium-skilled labor. *Economic Research Journal* **2021,** 56, (05), 91-108

15. Cai Yuezhou, M. W., How Data Influence High-quality development as a Factor and the Restriction of Data Flow. *Journal of Quantitative & Technological Economics* **2021,** 38, (03), 64-83.http://10.13653/j.cnki.jqte.2021.03.002.

16. Meng An, C. Z., Can the development of digital economy improve the efficiency of China's regional innovation. *Journal of Southwest Minzu University(Humanities and Social Sciences Edition)* **2021,** 42, (12), 99-108

17. Huixin Yang, H. T., Advancing China's manufacturing technology and climbing global value chain from perspective of coupled interaction. *Journal of Anhui University(Philosophy and Social Sciences Edition)* **2020,** 44, (06).http://10.13796/j.cnki.1001-5019.2020.06.016.

18. Huaichao Chen , X. T., Jianhong Fan, The interactive relationship among digital economy, digital literacy of talents and the upgrading of manufacturing structure: A PVAR analysis based on provincial Panel data. *Science & Technology Progress and Policy* **2022,** 39, (19), 49-58

19. Eshet-Alkalai, Y., Digital Literacy: A Conceptual Framework for Survival Skills in the Digital era. *Journal of educational multimedia and hypermedia* **2004,** 13, (1), 93-106

20. AUTOR D H, D. D., The growth of low-skill service jobs and the polarization of the US labor market *American Economic Review* **2013,** 103, (5), 1553-1597

21. Zhou, Y., Human capital, institutional quality and industrial upgrading: global insights from industrial data. *Economic Change and Restructuring* **2018,** 51, (1), 1-27.http://10.1007/s10644-016-9194-x.

22. Zhonglin Wen. Lei Zhang, Testing and application of the Mediating effects. *Acta Psychologica Sinica* **2004**, (05), 111-117

23. Zhanxiang, F. Y. Y. X. W., Structure Changes in Manufacturing Industry and

Efficiency Improvement in Economic Growth. *Economic Research Journal* **2016,** 51, (08), 86-100

24. Yuanhai Fu , X. Y., Zhanxiang Wang, Technological progress path selection for manufacturing structure optimization: Empirical analysis based on dynamic panels *China Industrial Economics* **2014**, (09), 78-90.http://10.19581/j.cnki.ciejournal.2014.09.006.

25. Jie Zhang, Z. C., Shufeng Wu, Wenhao Sun, Foreign technology iIntroduction and independent innovation of Chinese local enterprises. *Economic Research Journal* **2020,** 55, (07),

26. Qiannari, S., Development Pattern: 1950-1970. In Beijing: China Financial and Economic Publishing House, 1989.

27. Chen Huang, D. Q., STUDY ON THE INTERACTION BETWEEN URBANIZATION AND MANUFACTURE STRUCTURE UPGRADE——Based on Crowd in-out Effect. *Economic Theory and Business Management* **2017**, (05), 102-112

28. Donghua Yu , K. Z., Factor Market Segmentation,Technological Innovation Ability and Transformation and Upgrading of Manufacturing Industry. *East China Economic Management* **2020,** 34, (11), 43-53.http://10.19629/j.cnki.34-1014/f.200421003.

Figure

Click here to access/download;Figure;Figures.docx

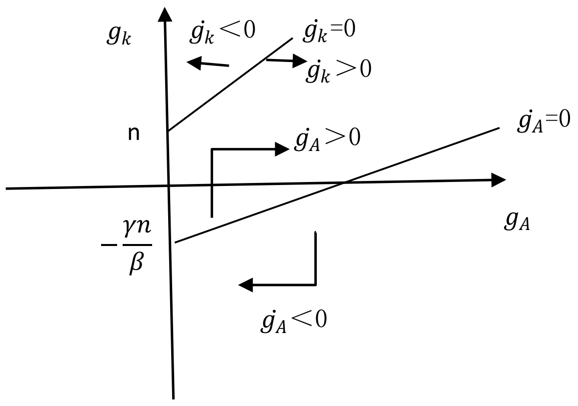

Figure 1 Capital and Knowledge Growth Rate Curve

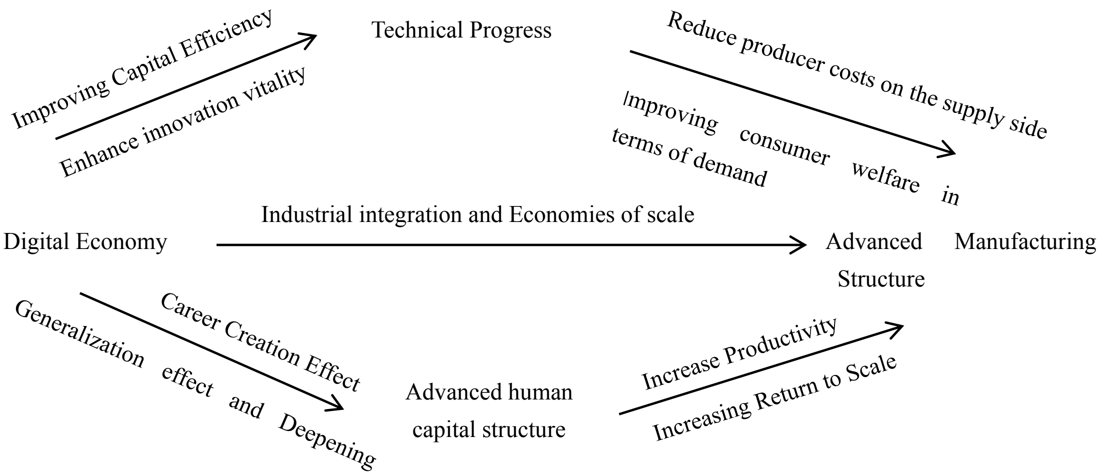

Figure 2: The Transmission Path of the Impact of Digital Economy on the Advanced Structure of Manufacturing Industry

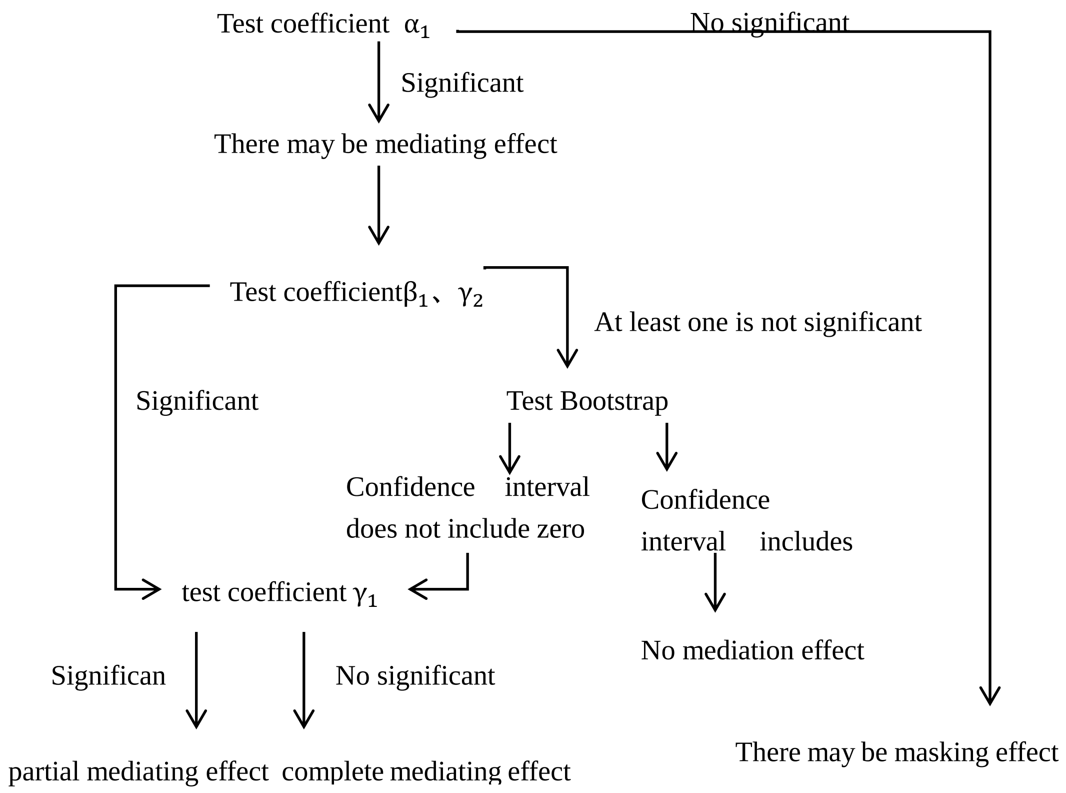

Figure 3：Diagram of mediating effect

Click here to access/download
Supporting Information
S1_File.pdf
